# What would happen if?: A comparison of fathers’ and mothers’ questions to children during a science activity

**DOI:** 10.3389/fpsyg.2023.1078994

**Published:** 2023-02-16

**Authors:** Kathryn Leech, Ian L. Chandler-Campbell, Jenna Alton, Kathleen H. Corriveau

**Affiliations:** ^1^School of Education, University of North Carolina at Chapel Hill, Chapel Hill, NC, United States; ^2^School of Behavioral and Brain Sciences, The University of Texas at Dallas, Richardson, TX, United States; ^3^Department of Human Development and Quantitative Methodology, University of Maryland, College Park, MD, United States; ^4^Wheelock College of Education and Human Development, Boston University, Boston, MA, United States

**Keywords:** scientific discourse, museum, parent–child interaction, questions, parent gender

## Abstract

Parents’ questions are an effective strategy for fostering the development of young children’s science understanding and discourse. However, this work has not yet distinguished whether the frequency of questions about scientific content differs between mothers and fathers, despite some evidence from other contexts (i.e., book reading) showing that fathers ask more questions than mothers. The current study compared fathers’ and mothers’ questions to their four- to six-year-old children (*N* = 49) while interacting with scientific stimuli at a museum research exhibit. Results indicated that fathers asked significantly more questions than mothers, and fathers’ questions were more strongly related to children’s scientific discourse. Results are discussed in terms of the importance of adult questions for the development of children’s scientific understanding as well as broadening research to include interlocutors other than mothers.

## Introduction

Children acquire knowledge about their world through interactions with adults such as parents (Vygotsky, 1978; Bruner, 1983). This may be especially true in scientific domains where such knowledge is often abstract or unobservable (Harris et al., 2006; Corriveau and Harris, 2014). In particular, parental talk about science promotes children’s own ability to talk and reason about scientific causal relations and mechanisms (Leinhardt and Crowley, 1998; Crowley et al., 2001a,b; Callanan et al., 2002; Jipson and Callanan, 2003; Canfield and Ganea, 2014). This study examines parent and child scientific discourse, and specifically whether differences exist between mothers’ and fathers’ use of questions about science. Below we highlight our rationale for focusing on questions within science conversations and potential differences between mothers and fathers before turning to our study design.

Parental questions, in particular wh-questions (e.g., *why did that happen?*), scaffold children’s development (Boland et al., 2003; Haden, 2010; Cristofaro and Tamis-LeMonda, 2012; Rowe et al., 2017). Compared to close-ended questions which can be answered with yes/no responses (e.g., *is the light on?*), wh-questions invite the child to continue the discussion and oftentimes engage in reasoning. Most studies have examined parental questions during book-reading and toy play (e.g., 3-bags task; Love et al., 2005) in relation to children’s language development. For example, parental wh-questions during contexts such as free play and reading are more likely to receive a child response than close-ended questions and are predictive of young children’s vocabulary development (Pancsofar and Vernon-Feagans, 2006; Pancsofar et al., 2010; Leech et al., 2013; Rowe et al., 2017).

In addition to language outcomes, there is accumulating evidence that wh-questions foster children’s conceptual and scientific knowledge. Ash (2004) documented the types of questions that move parent–child conversation toward a higher level of scientific understanding by qualitatively examining three families’ interactions at a science museum. Her findings suggest that questions that invite child explanations, are framed in an open-ended way, or build on prior conversations are most effective in promoting children’s scientific discourse. During science interactions, parent–child conversations containing wh-questions are longer and more sustained (Benjamin et al., 2010), relate to better child memory and recall of the scientific principles discussed while engaged in the exhibit (Hedrick et al., 2009; Benjamin et al., 2010; Haden et al., 2014), and increase child scientific discourse (Callanan et al., 2017; Eberbach and Crowley, 2017).

In the current manuscript, we add to this literature by exploring the relation between parents’ wh-questions and preschool children’s scientific discourse. We focus on children’s scientific discourse because prior research has indicated that children’s scientific explanations – specifically causal and mechanistic explanations – are related to children’s inductive inferences (Walker et al., 2014), generalizations (Legare and Lombrozo, 2014), and learning (Kurkul et al., 2021). Thus, it is important to explore how parental question-asking can prompt such science talk in young learners.

Our data were collected in a science museum which has several advantages including increasing access to both mothers and fathers. Traditionally, the literature on parent–child conversations has focused on children’s interactions with mothers, given their historical role as children’s primary caregiver. However, current demographic data in the United States indicate fathers play a considerably larger role in their children’s development than in previous generations (e.g., Cabrera et al., 2018). An examination of potential differences between mothers’ and fathers’ questions in science contexts is warranted because interactional differences have been found in non-science contexts. During book reading and toy play, fathers, on average, ask more wh-questions than mothers (Rondal, 1980; McLaughlin et al., 1983; O'Brien and Nagle, 1987; Leaper et al., 1998; Rowe et al., 2004, although see Tenenbaum and Leaper, 2003 and Pancsofar and Vernon-Feagans, 2006 for reverse patterns). We are not aware of direct comparisons of mothers versus fathers wh-questions in scientific contexts.

This study describes data on conversations in a museum between four- to six-year-old children and their father or mother. Unlike some museum research which focuses on large-group interactions (e.g., Diamond, 1986; Allen and Gutwill, 2009; Gutwill and Allen, 2010), or examines language at the level of the conversation (e.g., Crowley et al., 2001b; Pattison and Dierking, 2019), we coded and analyzed every verbal utterance within dyadic parent–child conversations. The first research question examined potential differences in the frequency of wh-questions between mothers and fathers. To our knowledge, no studies have examined differential rates of questioning between mothers and fathers in science contexts. We predicted that fathers would ask more wh-questions than mothers based on meta-analysis data showing that fathers ask more wh-questions than mothers in non-science contexts (Leaper et al., 1998). We also anticipated that interactional differences between mothers and fathers might not be limited to wh-questions, but to additional features of parental talk. Thus, we also examined possible differences in mothers’ and fathers’ use of close-ended questions and statements (i.e., non-questions).

The second research question examined the relation between parental questions and children’s scientific discourse. We predicted that children’s scientific discourse would be positively associated with parental wh-questions. Because underlying scientific mechanisms are often invisible, we argue that wh-questions may be a particularly effective strategy for fostering children’s scientific discourse. We also predicted that the frequency of parental wh-questions would be more strongly related to child scientific discourse than frequency of close-ended questions. Follow-up analyses examined whether these predictions were supported within both mother–child and father–child conversations.

In preliminary analyses, we explored child age and gender as potential covariates. Parents ask more questions to younger versus older children (Callanan et al., 2017), and talk more with “novices” versus “experts” (e.g., Palmquist and Crowley, 2007). Research indicates that the content of scientific conversations may also vary by child gender, for instance, explaining concepts more often to boys than girls (Crowley et al., 2001b) or using more challenging scientific language with adolescent boys (Tenenbaum and Leaper, 2003; Tenenbaum and May, 2014). Unfortunately, this study did not have adequate power to examine statistical interactions between child and parent gender, though we acknowledge this would be a fruitful topic for future research (see Crowley et al., 2001b).

## Materials and methods

### Participants

The sample included 49 English-speaking parent–child dyads recruited at a science museum in a large Northeastern city in the United States. Children (21 girls, 28 boys) were approximately 5 years, 5 months (*M*_age_ = 5.43 years, range = 4.00–6.91 years). Twenty-two mother–child dyads and 27 father–child dyads participated. Fourteen father–daughter, 13 father–son, 14 mother–daughter and 8 mother–son dyads comprised the sample. Ethnicity information was not collected for individual participants due to museum guidelines, but demographic information from the museum indicates it serves primarily European American families (Soren, 2009). Parents reported earning slightly higher than a bachelor’s degree (Mean years of education = 17 years; SD = 1.85; Range = 12–20). There were no differences between mother (*M* = 17.14; SD = 2.24) and father (*M* = 17.23; SD = 1.50) educational attainment, *t*(45) = 0.16, *p* = 0.87. Further, there was no difference between mothers and fathers in STEM-related (e.g., engineer) or non-STEM-related (e.g., letter carrier) occupations, *χ*^2^(1, *N* = 43) = 1.36, *p* = 0.24. This study was approved by both the institution and museum ethics review boards.

### Procedure

The data for this study were drawn from a larger study exploring a science learning intervention between parent–child dyads (see Chandler-Campbell et al., 2020). Here, we compare baseline data to explore potential differences parent-children science talk. Researchers approached families visiting the museum who appeared to have children in the study age range. If dyads agreed to participate, they were brought to a reserved corner of the museum’s exhibit floor.

#### Semi-structured parent–child interaction

Data analyzed in the current paper come from semi-structured parent–child interactions with a balance scale. The scale contained with two bins balancing on each side and approximately 75 differently colored toy bears which could be placed in either bin. Dyads were invited to play with the scale together as they would typically do at home. The researcher sat to the side of the table, let the dyad play, and did not interrupt until the parent or child reported they were finished. All interactions were dyadic, that is, between the target child and parent. The semi-structured interaction was videotaped for later transcription and coding. After the interaction, parents completed a paper-and-pencil survey in which they indicated their educational attainment and current occupation.

### Transcription and coding of parent–child conversation

All parent and child speech from the videos was transcribed verbatim by research assistants trained to reliably use the CHAT conventions of the Child Language Data Exchange System (CHILDES; MacWhinney, 2000). Each transcript was then independently verified by a second trained research assistant. The unit of transcription was the utterance, defined as any sequence of words that is preceded or followed by a change in speaker, intonation, or a pause. This process yielded 3,685 intelligible utterances across the entire sample, 2,407 of which came from parents (65.3 percent).

#### Parental question and statement coding

Trained research assistants coded each parent utterance for whether it was a wh-question, close-ended question, or statement. Every parent question utterance that was related to the balance scale activity was coded. We excluded any questions that were categorized as off-topic (*n* = 44; e.g., *what should we do later today?*).

##### Wh-questions

Question utterances that were framed with *who*, *what*, *when*, *where*, *why*, or *how* were coded as wh-questions (e.g., *what would happen if we put more bears on the left side? How does that work?*), the definition of which was adapted from Leech et al. (2013) and Rowe et al. (2004).

##### Close-ended questions

All remaining on-topic questions (e.g., *does it work? are you going to put that on?*) were coded as close-ended questions.

##### Statements

The number of statements, that is, non-questions, produced by parents (e.g., *this bin is heavier than this bin*.) was computed. Statements were counted by subtracting the number of questions from the total number of utterances produced. Total utterances were counted using automated analyses within CLAN (MacWhinney, 2000). Therefore, the sum of parent wh-questions, close-ended questions and statements reflect the total number of intelligible parental utterances produced during the interaction.

#### Scientific content coding

Parent and child utterances were also coded for references to scientific content. Two categories of coding were used: scientific and procedural, though analyses in this paper focus only on scientific codes. Scientific codes were defined as those that referenced a scientific fact (e.g., *how many bears are in this box? this is heavier than that bin*.) or causal process (e.g., *why is this bin heavier than this one; if you keep adding to this side it will go lower*). We coded utterances that made reference to balance, weight, or gravity, which were the scientific mechanisms inherent to the balance scale activity. Procedural utterances were defined as those which did not reference a scientific fact or mechanism; most were references to actions or directives (e.g., *put this over here; what one should we put in next?*).

#### Coding reliability

A team of research assistants was trained to implement the coding schemes described above. Research assistants were trained by coding 15 percent of the transcripts, which were compared to a gold standard set of codes prepared by the first two authors of the study. Once research assistants reached an acceptable level of reliability (Kappa > 0.70), they proceeded to code independently. Discrepancies in coding decisions were resolved through discussion between research assistants, and when necessary, a third coder was consulted. Coders were blind to study hypotheses and parent gender: transcripts did not mark whether the parent was a mother or father. Question and statement coding reliability averaged 95% (Cohen’s Kappa = 0.90). Scientific content coding reliability averaged 88% with a mean Cohen’s Kappa value of 0.75.

### Measures

#### Time on task

Unlike laboratory studies in which parent–child interactions typically take place during a fixed amount of time, we allowed dyads to engage with the balance scale for an open-ended amount of time. Therefore, we calculated *time on task*, or the number of minutes that dyads engaged with the scale after the experimenter introduced the task.

#### Parent question and statement utterances

The total numbers of *wh-questions*, *close-ended questions* and *statements* were calculated for each parent using the CLAN program. Rates (utterances per minute) were also calculated to control for differences in time on task.

#### Parental scientific utterances

The CLAN program calculated *parents’ scientific talk* by tallying the number of utterances that received a scientific code. We also identified and tallied utterances that received both a scientific code and a wh-question code, yielding a measure of *parents’ scientific wh-questions*. Scientific talk variables were also converted into rates (utterances per minute) to control for differences in time on task.

#### Children’s scientific utterances

Children’s scientific utterances were calculated as the total number of child utterances that received a *scientific* code. We chose to collapse children’s scientific questions and statements together because the majority of child scientific utterances were statements (*M* = 8.44; SD = 7.85) rather than questions (*M* = 0.43; SD = 0.93).

### Analysis plan

First, preliminary analyses examined whether key language variables differed as a function of child age and gender. Next, descriptive statistics for each parent talk variable was reported along with their inter-correlations. Finally, Poisson regression was used to compare mothers’ and fathers’ use of questions and the relation between parental talk and children’s scientific discourse.

Poisson regression was used because there was significant variation in time on task across dyads, with the average dyad spending approximately 4 min (*M* = 3 min 52 s; SD = 1 min 40 s), although the range extended from 1 min 14 s to 8 min 50 s. Father–child dyads (*M* = 4.33 min; SD = 1 min; 40 s) interacted with the activity significantly longer than mother–child dyads (*M* = 3.33 min; SD = 1 min; 35 s), *t*(48) = 2.14, *p* = 0.03. Because of this difference, data were modeled using Poisson regression with time on task as an offset, which allowed us to model the rate of utterances observed per minute rather than the number of utterances used per participant. This ensured that any effect of parent gender on question use was not due to differences how long the dyad engaged with the activity. Offsets are an appropriate choice when the time period during which particular behaviors occur is not consistent across the sample (Gelman and Hill, 2006). When deciding on the appropriate offset, we considered both the total number of utterances, which is typical of other semi-structured protocol such as the three-bag task, and time on task. We chose the latter because the interaction was open-ended in terms of time, and any differences in time would in turn influence the total number of utterances.

Checks of model fit revealed evidence of over-dispersion, a violation of the Poisson assumption that the variance is equal to the mean. We refit models with quasi-Poisson distributions to allow for over-dispersion when necessary (Hardin et al., 2007). Over-dispersion can lead to biased standard error estimates, and the quasi-Poisson distribution corrects for this violation by widening standard error estimates for all predictors. All analyses were run using the glm2 package (Marschner, 2011) in R.

## Results

### Preliminary analyses

Preliminary analyses examined associations between child age, child gender, and key parental language variables (wh-questions, close-ended questions, and statements). No significant correlations emerged and thus we did not consider child gender or age as covariates in subsequent analyses.

### Descriptive patterns of parent–child conversation

Table 1 presents descriptive statistics for each parent talk variable and their inter-correlations. On average, parents in this sample asked 19 questions and produced 27 statements during the balance scale activity. Parents asked significantly fewer wh-questions than close-ended questions, *t*(48) = 4.76, *p* < 0.001. A similar pattern of parents’ question-asking emerged when question variables were considered as rates per minute (right portion of Table 1, *Frequencies per Minute*). Table 2 displays descriptive statistics (raw frequencies and rates per minute) for all conversational variables for mothers and fathers separately.

**Table 1:** Descriptive statistics of parent language codes (*N* = 49).

Variable	Raw frequencies of utterances						Rate of utterances per minute					
	Mean (SD)	Range	1	2	3	4	Mean (SD)	Range	1	2	3	4
1. Wh-	6.53 (5.10)	0.00–18.00	–				1.57 (1.12)	0.00–4.31	–			
2. Close-ended	12.59 (11.16)	0.00–52.00	0.63^***^	–			3.10 (2.06)	0.00–8.52	0.39^**^	–		
3. Total questions	19.12 (14.89)	1.00–63.00	0.81^***^	0.96^***^	–		4.67 (2.70)	0.38–10.33	0.71^***^	0.93^***^	–	
4. Total statements	27.06 (17.12)	3.00–76.00	0.46^**^	0.48^**^	0.43^***^	–	6.84 (3.47)	1.94–18.31	0.13	0.25	0.24	–

^**^p < 0.01; ^***^p < 0.001.

**Table 2:** Descriptive statistics of parent–child conversation variables for mother–child and father–child interactions.

	Raw frequencies of utterances				Rate of utterances (per minute)			
	Mother–child		Father–child		Mother–child		Father–child	
	Mean (SD)	Range	Mean (SD)	Range	Mean (SD)	Range	Mean (SD)	Range
Parent wh-questions	4.23 (3.95)	0–11	8.41 (5.13)	0–18	1.16 (1.05)	0–4.31	1.90 (1.06)	0–4.04
Parent scientific wh-questions	2.14 (2.46)	0–7	4.70 (3.71)	0–12	0.60 (0.61)	0–1.96	1.05 (0.82)	0–2.96
Parent close-ended questions	10.50 (10.50)	0–52	14.30 (11.40)	0–43	3.04 (2.14)	0–8.52	3.15 (2.00)	0–7.02
Parent statements	21.20 (13.00)	3–46	32.00 (18.60)	6–76	6.36 (3.54)	1.94–14.40	7.23 (3.42)	3.02–18.30
Parent scientific utterances	13.00 (20.10)	0–118	15.60 (12.30)	0–51	4.21 (3.62)	0–14.10	4.77 (3.55)	0–17.60
Child scientific utterances	5.30 (5.35)	0–16	11.50 (8.74)	0–33	1.88 (1.56)	0–5.45	2.74 (2.38)	0–11.00

### Comparing mothers’ and fathers’ use of questions

Poisson regression models examined whether use of wh-questions, close-ended questions, or statements varied between mothers and fathers (Table 3). Model parameters reflect the rate of utterances (per minute), but all findings held using the raw frequency of utterances. The only feature of parent talk found to significantly differ between mothers and fathers was wh-questions: fathers’ rate of wh-questions (*M* = 1.90 questions per minute) was nearly twice the rate of mothers’ (*M* = 1.15 questions per minute). The rate of close-ended questions did not vary by parent gender, nor did the rate of statements.

**Table 3:** Poisson regression analyses for rate of questions and statements by parent gender.

	Parent talk variable (rates per minute)		
	Wh- *B* [95% CI]	Close-ended *B* [95% CI]	Statements *B* [95% CI]
Intercept	0.23 [−0.09, 0.53]	1.14^***^ [0.82, 1.43]	1.84^***^ [1.60, 2.06]
Parent Gender	0.43^*^ [0.06, 0.81]	0.05 [−0.34, 0.44]	0.15 [−0.13, 0.44]

^**^p < 0.01; ^***^p < 0.001. Parent gender was dummy coded such that 0 = mother and 1 = father.

### Relation between parental talk and children’s scientific discourse

**Figure 1:**
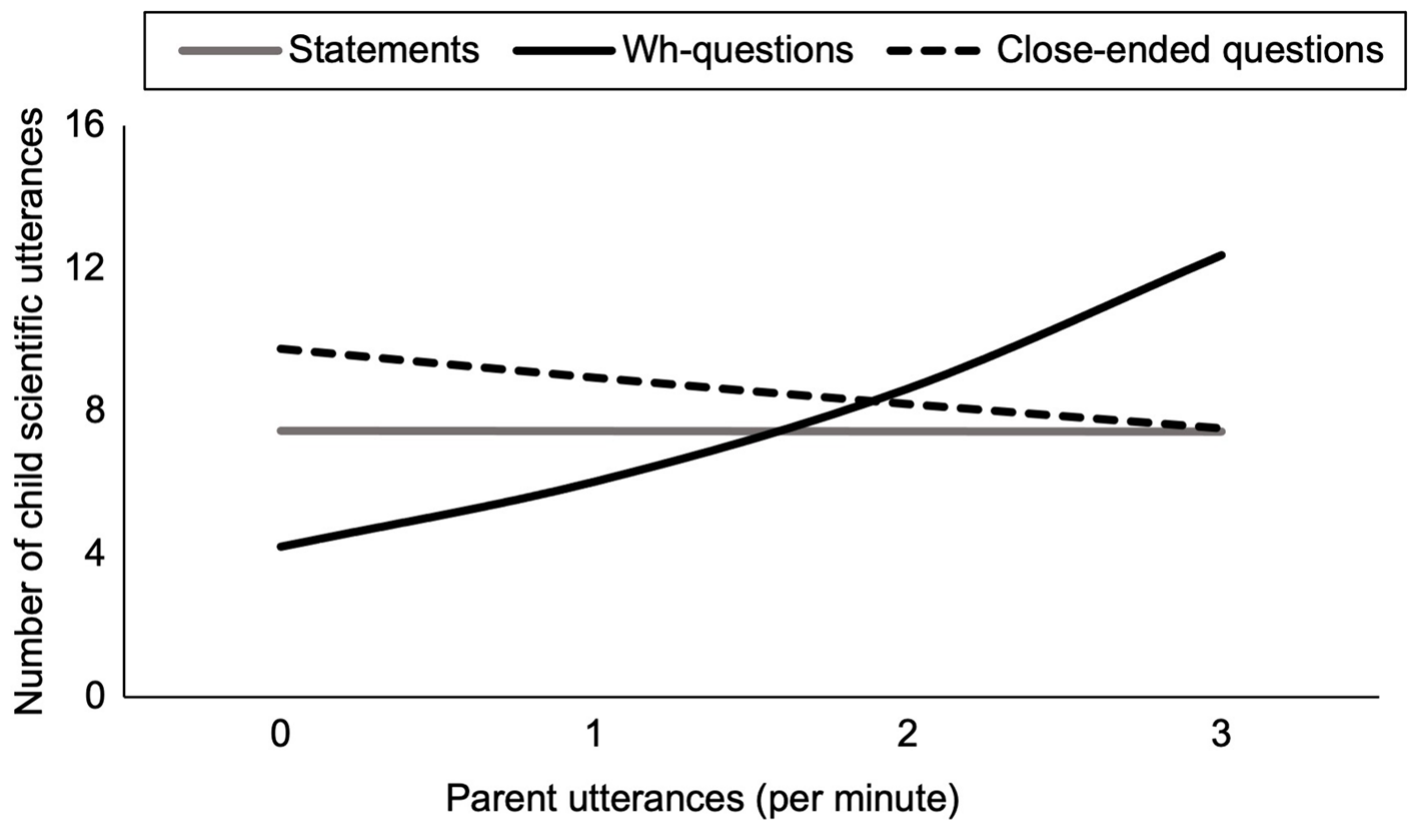
Parents’ wh-questions are positively associated with children’s scientific discourse (solid black line), whereas the effect of close-ended questions on child discourse is negative but non-significant (dashed black line). There was no association between parental statements and children’s scientific discourse (grey line). Figure depicts estimates which were derived from Table 4, Model 1 using the Effects package in R.

Next, we examined whether wh-questions, close-ended questions, or statements were associated with children’s scientific discourse, and if so, whether parent gender moderated these relations. *N* = 2 children were not included in this analysis as their scientific talk was more than three standard deviations above the sample mean. Further inspection revealed that the inflated measures of scientific talk from these children were due to the majority of the time spent counting the elements to be placed on the balance scale. Table 4 presents a series of Poisson regression models predicting children’s scientific discourse from main effects of wh-questions, close-ended questions, and statements, and their interaction terms with parent gender. Parental speech variables were entered in the same model for parsimony, as their correlations were non-significant or weak (Table 1). In Model 1 (Table 4), the positive coefficient for wh-questions per minute indicates that a higher rate of parent wh-questions was associated with more child scientific talk (Figure 1). Close-ended questions and statements were not significantly associated with child scientific talk.

**Table 4:** Series of regression modeling predicting children’s scientific discourse (*N* = 47).

	Model 1 *B* [95% CI]	Model 2 *B* [95% CI]	Model 3 *B* [95% CI]
Intercept	0.47 [−18, 1.09]	−0.50 [−0.43, 1.37]	0.96^*^ [0.14, 1.71]
Wh-q per min.	0.36^**^ [0.15, 0.57]	0.33^**^ [0.10, 0.56]	0.003 [−0.42, 0.37]
Close-q per min.	−0.09 [−0.20, 0.02]	−0.12 [−0.36, 0.07]	−0.10~ [−0.21, 0.01]
Statements per min.	−0.001 [−0.07, 0.06]	−0.01 [−0.08, 0.06]	−0.01 [−0.08, 0.05]
Parent (father)		0.04 [−0.84, 0.97]	−0.63 [−1.51, 0.26]
Parent X Close-q per min.		0.06 [−0.19, 0.32]	
Parent X Wh-q per min.			0.50^*^ [0.05, 0.98]
Adjusted R^2^ (%)	31.1	31.2	41.3

~*p* < 0.10; ^*^p < 0.05; and ^**^p < 0.01.

#### Does parent gender moderate the effect of wh-questions on children’s scientific discourse?

**Figure 2:**
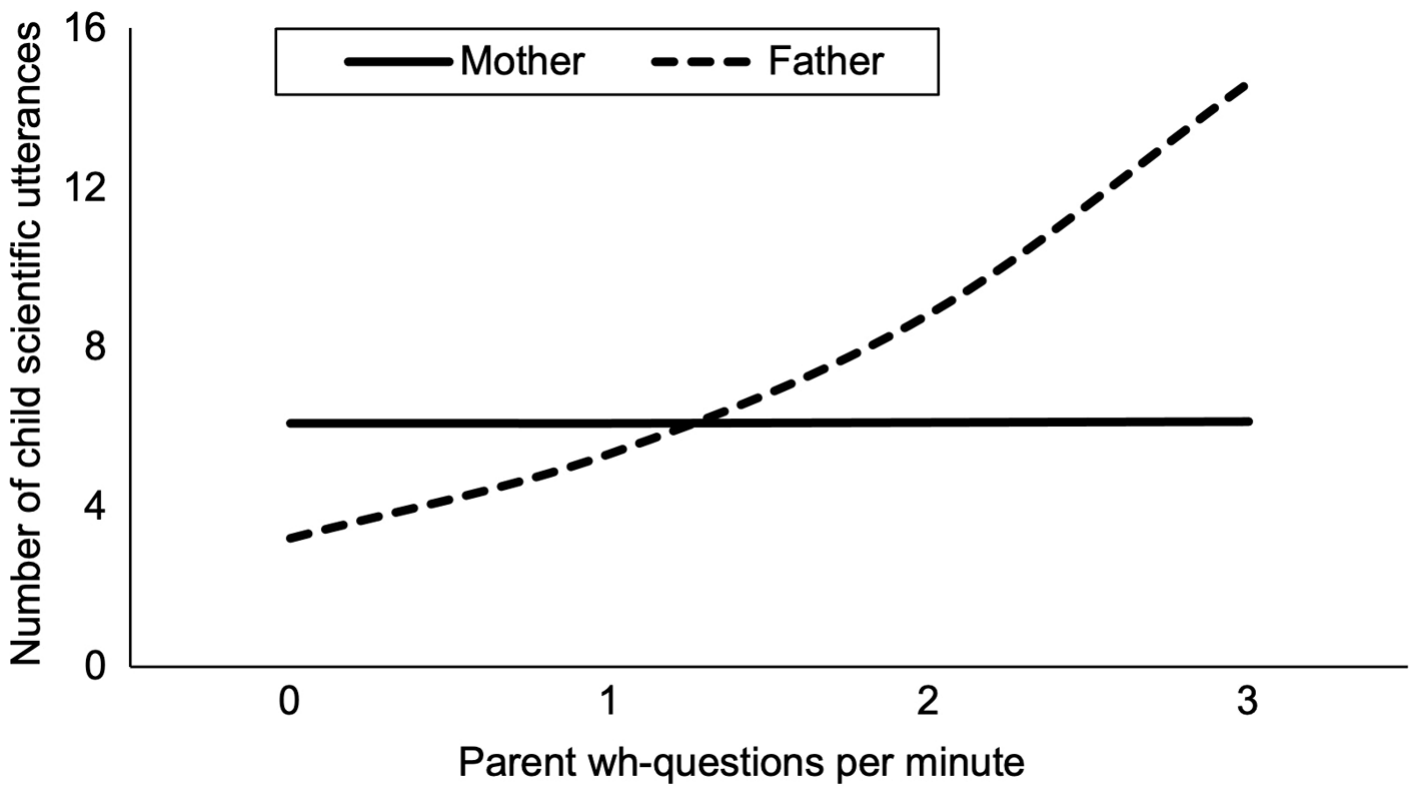
Estimated effect of parent wh-questions on children’s scientific discourse. Figure indicates that this relation is moderated by parent gender such that fathers’ questions are significantly and positively related to children’s talk whereas mothers’ questions are not. Estimates were derived from Table 4 Model 3 using the Effects package in R.

Table 4 (Model 2) shows that parent gender did not moderate the effect of close-ended questions on children’s scientific talk (Table 4, Model 2). That is, the non-significant association between close-ended questions and children’s scientific discourse was observed for both mother–child and father-child interactions. However, the significant interaction term in Table 4 (Model 3) reveals that parent gender moderated the effect of wh-questions on children’s scientific talk. A follow-up simple slopes analyses suggested that fathers’ questions positively related to children’s scientific discourse (*t* = 3.57, *p* = 0.001), whereas mothers’ questions did not, *t* = 0.53, *p* = 0.60 (Figure 2).

### Why are fathers’ but not mothers’ wh-questions related to child scientific discourse?

Though not an *a priori* research question, we performed two exploratory analyses to better interpret the interaction between parents’ wh-questions and gender. We analyzed (1) whether there was a difference in children’s likelihood of providing an immediate response to father versus mother wh-questions, and (2) if fathers’ wh-questions contained more scientific content than mothers’ wh-questions.

First, to determine the role of child responses, we recoded every parental question (both wh-questions and close-ended questions) to reflect whether it received an immediate response from the child. Immediate responses were defined as a child verbal turn that followed directly from a parental utterance. Of the 937 parent questions, 348 questions received an immediate response from children (37 percent). Of those responses, 45 percent (*n* = 155 responses) were coded as scientific, 26 percent of children’s total scientific utterances. To determine the likelihood of an immediate child scientific response, we fit a multilevel logistic regression model with adult question as the unit of analysis and participant as a random effect. In the model, we included question type (wh-, close-ended), parent gender, and their interaction term as predictors. Model results indicated a significant main effect for question type, *B* = 1.43; *z* = 5.95, *p* < 0.001. That is, the likelihood of a child scientific response was significantly higher for wh-questions than for close-ended questions, controlling for whether children were interacting with mothers or fathers. The main effect of parent gender was not significant, nor was the interaction term between question type and parent gender, *B* = −0.39; *z* = −0.74, *p* = 0.46. These latter effects suggest that in this sample, both mothers’ and fathers’ wh-questions were equally likely to elicit immediate scientific responses from children.

Second, we considered the possibility that fathers’ wh-questions contained more scientific content than mothers’, therefore prompting more scientific talk from children. To explore this possibility, we compared the number of scientific wh-questions across parent gender (see Table 5 for examples from the corpus). Parents, on average, asked 3.45 scientific wh-questions (SD = 3.49; Range = 0 to 12), comprising roughly 9.4 percent of their total utterances. Regression analysis confirmed that fathers asked significantly more wh-questions (*M* = 1.05 per minute; 11.0 percent of utterances) containing scientific content compared to mothers (*M* = 0.60 per minute; 6.8 percent of utterances), *B* = 0.72; *t* = 2.02, *p* = 0.04, [95% CI: 0.05, 1.46]. Thus, in addition to using more overall wh-questions, fathers also produced more scientific wh-questions.

**Table 5:** Example scientific and procedural wh-questions from mothers and fathers.

	Scientific	Procedural
Mothers	1. Why do you think that is? 2. How many is that? 3. How do you think we can even that out?	1. What do you got in there? 2. What is it? 3. Now what should we do?
Fathers	1. Why is it like the balls? 2. How many would it take to level the that out? 3. If we keep putting them on this side what is that do?	1. What else can we do with this toy? 2. How do you want to do it this time? 3. Alright how can we do that?

The final step was to determine whether fathers’ scientific wh-questions were more strongly associated with children’s scientific discourse compared to mothers’ scientific wh-questions. Regression analysis revealed a significant interaction between parent gender and scientific wh-questions, such that fathers’ scientific wh-questions were more strongly associated with children’s scientific discourse than mothers’ questions, *B* = 0.55; *t* = 3.14, *p* = 0.001, [95% CI: 0.21, 0.89]. However, this model was over dispersed and once standard errors were corrected, the interaction effect only trended toward significance, *p* = 0.11. Thus, a conservative interpretation is that fathers’ wh-questions—both scientific and procedural—are associated with more scientific discourse from children.

## Discussion

This study examined children’s science conversations with parents, specifically focusing on question-answer exchanges. A main finding of this study was that parental wh-questions were positively associated with child scientific discourse, whereas close-ended questions were not. Importantly, these findings were qualified by interactions with parent gender: fathers asked significantly more wh-questions than mothers, and the positive relation between parental wh-questions and children’s scientific discourse was only found in interactions with fathers.

Our data indicated that children’s scientific discourse was positively associated with the rate of parent wh-questions (per minute), and children were significantly more likely to respond scientifically to a wh-question, as compared to a close-ended question. Why might a higher rate of wh-questions relate to more child scientific talk? Controlled experimental studies offer some clues. Consider the difference between the following two parent utterances taken from our corpus of parent–child conversations. These utterances convey the same content but differ in whether the utterance functions as a question (A) or statement (B).

- How can we test the scale to see if it is unbalanced?
- Let us test the scale by putting the same number of weights on both sides.

Yu et al. (2018) propose that although both (A) and (B) transfer knowledge to the child, (B) would constrain a child’s potential exploration and subsequent discussion about the scientific phenomenon. On the other hand, (A) expands the potential space of exploration and discussion about balance and weight between the parent and child. We argue that wh-questions during informal learning activities bring forward two situations that are known to scaffold children’s science discourse: directing children’s attention to important features of the activity (e.g., balance, weight), and prompting children to think and speak within their zone of proximal development (Vygotsky, 1978). Thus, it is plausible that although children may not always respond immediately to a question such as (A), the wh-question may lead to a subsequent scientific utterance later in the conversation. This framework is also useful in explaining may why close-ended questions were not related to children’s scientific talk: close-ended questions likely constrain children’s exploration and scientific talk to a similar degree as Yu et al. (2018) found with statements such as (B).

Although other studies point to the importance of parental wh-questions for children’s learning (Benjamin et al., 2010; Haden et al., 2014; Callanan et al., 2017), this is the first study to directly compare children’s responses to wh-questions versus close-ended questions as they occur around a scientific activity. Haden (2010) has argued that it is not the frequency with which parents ask questions, the more important aspect is *how* these questions promote learning. Our data supports this argument: in our sample, close-ended questions (*M* = 3.10 questions per minute) were two times more frequent than wh-questions (*M* = 1.57 questions per minute), yet close-ended questions were not related to children’s scientific discourse. Differential relations between wh- and close-ended questions to children’s talk holds important implications for educators and parents regarding how to facilitate children’s engagement in informal, and perhaps formal, scientific contexts. For instance, an adult who asks only a few wh-questions may confer larger benefits for their child’s engagement and learning compared to an adult who asks many close-ended questions.

The second major finding of this study was that fathers asked significantly more wh-questions than mothers. Results were presented in the rate of utterances (per minute) in order to control for differences in the length of time spent engaged in the activity. These results indicate that the density of fathers’ wh-questions was greater than that of mothers’, and this difference was not explained by the fact that fathers spent more time with children on the balance scale activity and therefore had more opportunities to ask questions.

This work is both similar to and different from prior work on mother and father conversation in non-scientific settings. For example, our work is consistent with Leaper et al.’s (1998) meta-analysis, indicating that one of the largest differences between mother and father interactions is use of wh-questions. Further, differences between mothers and fathers seems to be isolated: we only found that the rate of wh-questions differed, not close-ended questions or statements. This is similar to findings from Rowe et al. (2004) showing that only wh-questions differed between mothers and fathers, not total questions (which included close-ended questions). In contrast, however, other studies report that mothers ask more close-ended questions than fathers (Leaper et al., 1998; Tamis-LeMonda et al., 2012), which we did not observe in the current study.

These findings contribute to previous work showing that fathers’ wh-questions during book reading and toy play at home are related to various indices of language and cognitive development between 24- and 36-months (Leech et al., 2013; Rowe et al., 2017). Our study broadens our understanding of fathers’ challenging communicative style by showing these effects in other contexts such as the museum, with older children (i.e., 4- to 6-year-olds), and during interactions around scientific activities. Although parents in our sample were highly educated on average, previous work has found that fathers without a college degree also ask more wh-questions than mothers (Rowe et al., 2004). However, as questioning patterns vary by cultural context and reflect the broader socialization goals of that society (Schröder et al., 2013), it is important that generalizations of this study be limited to middle-class families in the United States.

Not only did fathers ask more wh-questions, but their wh-questions were more strongly associated with children’s scientific discourse. A post-hoc analysis offered one explanation for this finding: fathers’ wh-questions more often referenced scientific concepts, perhaps prompting children to engage in more scientific talk themselves. Of course, both speakers are co-constructing the conversation, and children are likely playing an important role in eliciting fathers’ questions. To that end, an additional explanation we did not explore in this paper is the contribution of children’s own interest and background knowledge of the topic. Children who demonstrate more interest in physical science may be initiating additional questions from parents, leading to extended back-and-forth conversation. Future research should explore the bi-directional associations between children’s science interest and parents’ language input. In addition, we did not explore the relation between scientific close-ended questions and children’s scientific discourse, as theory and empirical data point to open-ended questions as more strongly related to scientific discourse. Future studies may consider how the delivery of scientific information using close-ended questions or statements relate to children’s talk about science.

Fathers’ high rate of scientific wh-questions adds to previous findings that fathers tend to challenge children to converse and reason beyond their current ability level (Gleason, 1975). However, when looking more closely at the likelihood of a child response, fathers’ and mothers’ wh-questions were equally likely to elicit children’s scientific discourse. These results suggest that fathers’ and mothers’ questions are both an important element in supporting children’s scientific discourse, but that the frequency with which fathers engage in this conversational move is more frequent than mothers. Indeed, Benjamin et al. (2010) found no difference in the rate of father and mother wh-questions after an experimental manipulation that instructed parents to increase elaborative talk such as wh-questions. This suggests that interventions which focus on boosting wh-questions may be equally beneficial to both mothers and fathers.

Though we did not observe that mothers’ wh-questions related to child discourse, we must acknowledge other studies which have (e.g., Cristofaro and Tamis-LeMonda, 2012). One possibility beyond the scope of the present study is that mothers were using different conversational strategies than asking wh-questions while playing with the balance scale. For example, mothers have been found to engage in more supportive talk to children than fathers (Leaper et al., 1998). Thus, supportive talk may be positively influencing other aspects of the interaction, such as child interest or enjoyment, which were not measured outcomes in this study.

A limitation of this study is that the small sample size precluded us from potentially observing effects of both parent and child gender and their interactions. Although child gender was not significantly related to any parent or child conversational variables, it is possible that a larger sample size would have had the power to detect such effects. Furthermore, parent gender effects should be interpreted with caution due to the small sample size. Future work with larger samples should seek to replicate these findings as additional evidence of differences in maternal and paternal discourse patterns. A second limitation is that the current sample included a relatively small number of mother-son dyads. This was not by choice but reflected a recruitment decision to invite participation from any parent–child dyad visiting the museum who fell into the study age range. The unique and combined effects of parent and child gender are interesting and important and would be well-suited for a more controlled study outside of the museum where both parent and child gender are equally distributed.

An interesting direction for future research concerns whether the patterns of conversation around the balance scale—a physical science activity—would replicate in contexts that expose children to other scientific domains. There is evidence from the literature that conversational content varies based on the scientific domain of the activity: dyadic math and engineering talk is more common in science museum exhibits that focus on building, whereas biological science talk occurs frequently in settings such as aquaria and live animal exhibits (Rowe and Kisiel, 2012; Marcus et al., 2017; Kelly et al., 2022). Parent–child conversations in biological exhibits such as aquaria provide opportunities that the balance scale activity does not afford, such as talk about the life cycle and biological processes (Kelly et al., 2022). Touching and observing live animals in biological exhibits may also lead to opportunities for additional language interactions, such as comparing and contrasting and highlighting discrepancies (Rowe and Kisiel, 2012). However, there are likely conversational features that are common to all contexts, such as questions from parents, hypothesis testing, and a focus on general problem solving. Thus, future research should examine whether differences between mothers’ and fathers’ questioning patterns extend beyond the physical domain.

In summary, this study adds to existing evidence that parental wh-questions support children’s participation in science conversations. We extend this work by showing that fathers, on average, asked more questions, which are associated with more scientific discourse from children. Fathers’ strengths can serve as a unique and additive role to mothers in supporting children’s developing conceptions about science.

## Data availability statement

The raw data supporting the conclusions of this article will be made available by the authors, without undue reservation.

## Ethics statement

The studies involving human participants were reviewed and approved by Boston University IRB. Written informed consent to participate in this study was provided by the participants’ legal guardian/next of kin.

## Author contributions

KC and IC-C conceptualized the study design. IC-C, JA, and KL collected and coded the data. KL drafted the manuscript. KC, IC-C, and JA provided critical revisions. All authors contributed to the article and approved the submitted version.

## Funding

This work was funded by NSF Grants #1652224 and #1640816 to KC.

## Conflict of interest

The authors declare that the research was conducted in the absence of any commercial or financial relationships that could be construed as a potential conflict of interest.

## Publisher’s note

All claims expressed in this article are solely those of the authors and do not necessarily represent those of their affiliated organizations, or those of the publisher, the editors and the reviewers. Any product that may be evaluated in this article, or claim that may be made by its manufacturer, is not guaranteed or endorsed by the publisher.
